# Gripping adhesive principles in the design of effectors

**DOI:** 10.1007/s42452-022-05200-y

**Published:** 2022-10-22

**Authors:** Marcel Horák, Michal Starý, Ondřej Matúšek, František Novotný

**Affiliations:** Department of Glass Producing Machines and Robotics, Faculty of Mechanical Engineering, Technical University of Liberec, Studentská 1402/2, 461 17 Liberec 1 Liberec, Czech Republic

**Keywords:** Gripping element, Adhesion, Computer simulation, Cohesive energy, Vacuum, Position compensation

## Abstract

This article presents a basic study of knowledge in the research and development of specific gripping elements based on the principle of adhesion. It summarizes the use of materials with a high degree of surface adhesion in the design of gripping elements usable in industry to provide stable gripping of objects during automatic manipulation. The principle of a combined element proposed by the authors, where the gripping force is derived through both vacuum and adhesion, is presented. The conditions of operation in an active or completely passive mode without the need to connect an energy source are discussed in detail. In the active mode, a significant increase in gripping forces is demonstrated compared to standard vacuum elements, which has a positive effect on the amounts of compressed air consumed and the level of grip safety in production processes. To ensure the optimal function of the adhesive gripping elements, the design of a specifically designed fluid position compensator and an active system for disturbing the adhesive gripping forces is presented. The functionality of the designed element is demonstrated through several laboratory tests under various conditions, and the results clearly confirm an increase in gripping forces in the axial and in particular the radial direction of the load. The research includes the design of a computer model of deformation-adhesive contact, respecting the time dependence of the deformation of the adhesive layer and the gradual loss of contact with the object.

Article highlights:

o Experimental study presents use of PU materials in adhesive and combined gripping elements.
o Adhesive contact theory is applied for a numerical simulation and prepared computer model is subsequently verified.
o Authors present new proprietary solution of gripping element applicable in industrial robotics.

## Introduction

Despite the global crisis caused by the new type of coronavirus SARS-CoV-2, according to press releases published on the International Robotics Federation (IFR) website, up to 2 million new industrial robots are expected to be installed worldwide between 2020 and 2022 [1]. Applications in the shared workspace are increasingly being discussed, where collaborative types of robots are deployed, which now have a whole range of innovative solutions applying sensor fusion, compact types of drives, and control systems combined with intelligent software. The range of possibilities has expanded dynamically since 2006, when one of the first collaborative robots was introduced by KUKA, and new companies (Universal Robots, Techman Robot, On Robot, MiR, Fetch Robotics, etc.) investing in the research and development of robots that respond in real time to changes in the environment, gestures, voice, etc., have logically emerged. A clear trend is in line with the call of Industry 4.0 to provide a standardized communication interface between robots with Open Platform Communications (OPC), which can also be connected to peripherals, the Industrial Internet of Things (IIoT), and cloud technologies.

Modern technologies and applications of both industrial and service robots, whose numbers have increased by tens of percent year-on-year over the last few years, bring new demands and requirements on the end power members of the robots, known as effectors. A very important group of effectors are gripping heads [2, 3], which differ in the way the gripping force is applied. Standard heads are mechanical, vacuum, or magnetic, but there are also completely unique heads using new or innovative principles for gripping the manipulated objects. A typical example of a modern approach is the application of an electrostatic charge [4–6] (electro adhesion) or a high degree of surface adhesion of new materials in the design of gripping elements, heads, and grippers, which is presented below and represents an interesting alternative in terms of minimizing energy requirements and maximizing efficiency.

**Fig. 1:**
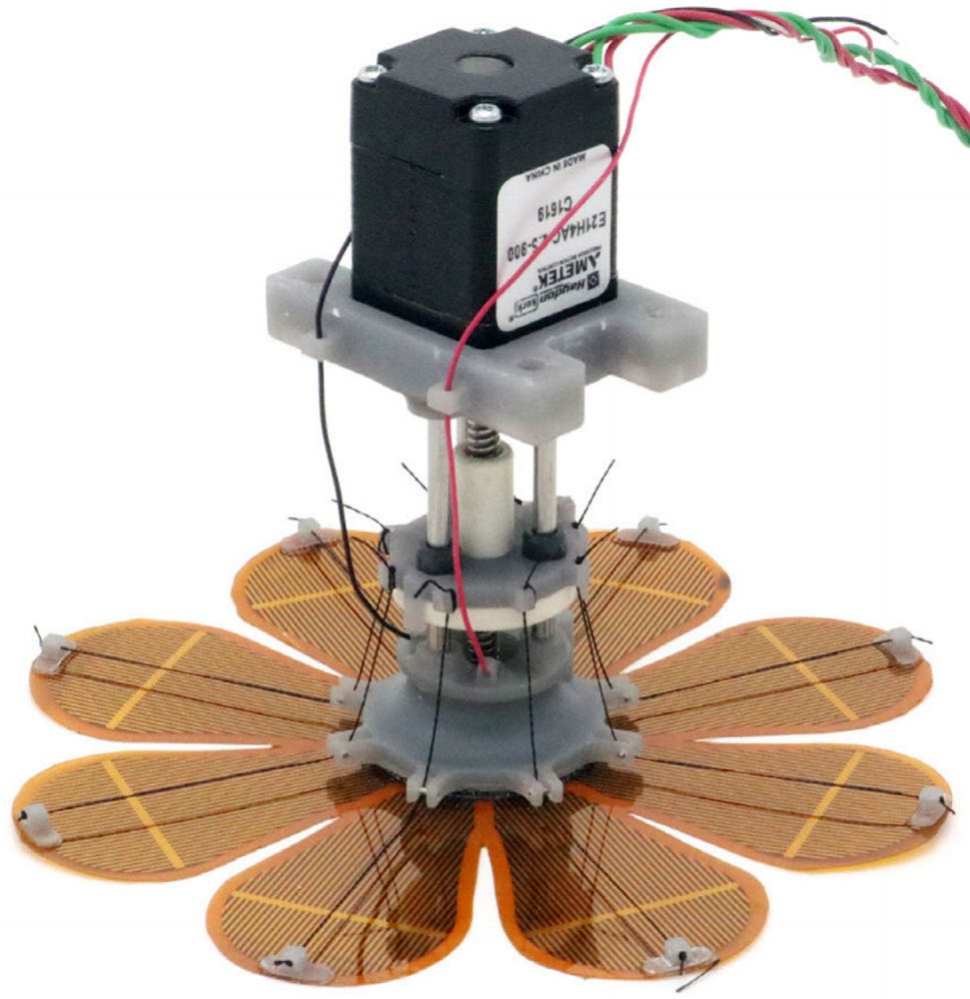
Example of the design of an electrostatic gripper [4]

Another observed parameter is the ability of grippers to react to changes in the shape of the manipulated objects as a result of today’s typical production of a wide range of products, which very often changes and is dependent on the current demand. Related to this is the research and development of soft grippers and gripping elements with a variable geometry of contact surfaces based on the principles of the memory effects of new materials. These include dielectric elastomers (Fig. 1), electro-active polymers, or ionic polymer metal composite actuators, which are summarized in [7, 8].

**Fig. 2:**
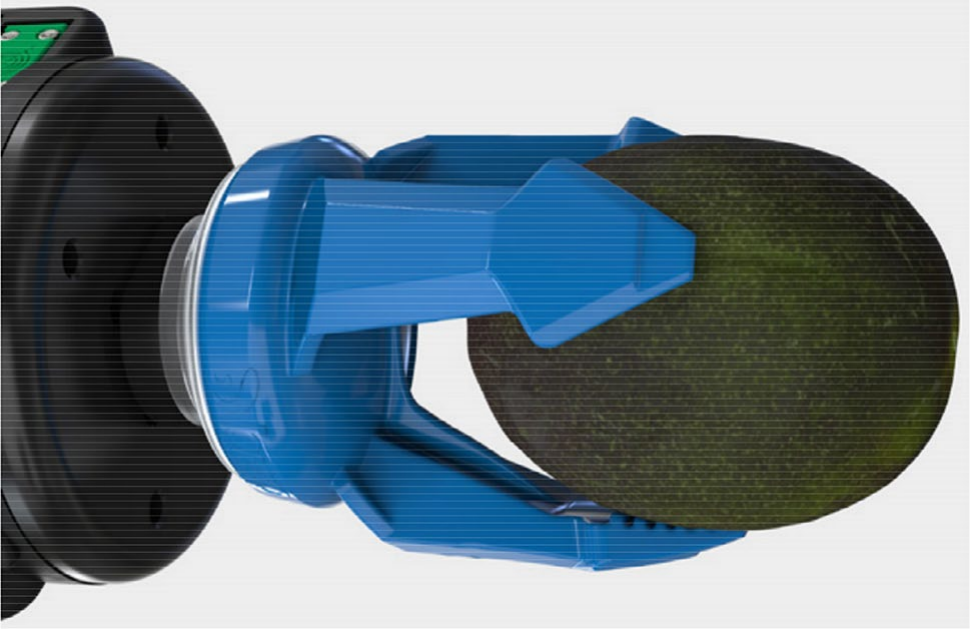
Soft industrial gripper based on the principle of a pneumatic muscle [11]

A separate group that has been tested for decades is represented by gripping elements that use fluid muscles (Fig. 2) with controlled (limited) deformation in the required direction [9, 10].

**Fig. 3:**
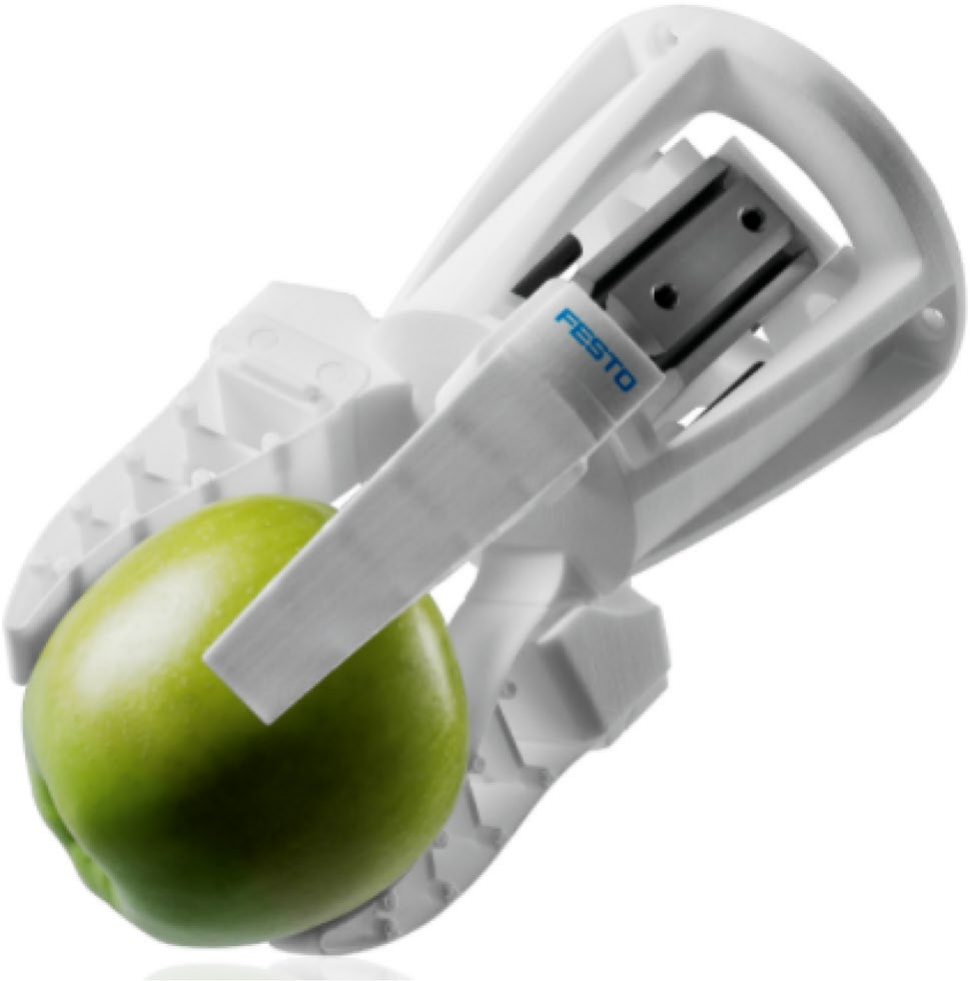
Mechanical gripper with passive shape-adaptive elements [12]

The classic approach is the application of passive (Fig. 3), or active shape-adaptive gripping elements combined with active parallel grippers, which are also increasingly being used in industry.

**Fig. 4:**
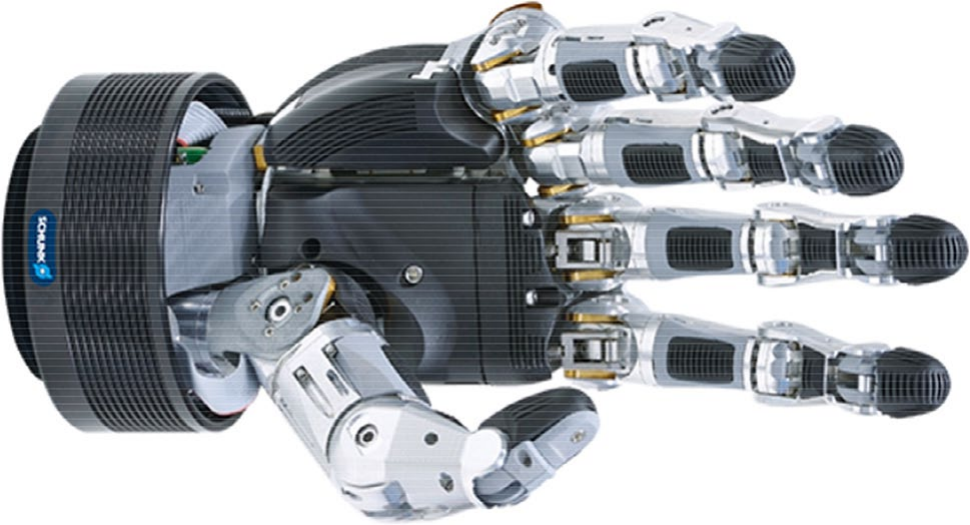
SVH anthropomorphic gripper [13]

Anthropomorphic heads copying the function of the human hand traditionally represent the state-of-the-art in the field of mechatronic grippers with integrated drives, sensors, and controls (Fig. 4), and have been standard in the production portfolio of renowned effector manufacturers for the last few years [13, 14]. However, there disadvantages include their high price of 45,000 EUR, low overload capacity, and relatively complicated control method, which has so far significantly limited their deployment in common applications.

**Fig. 5:**
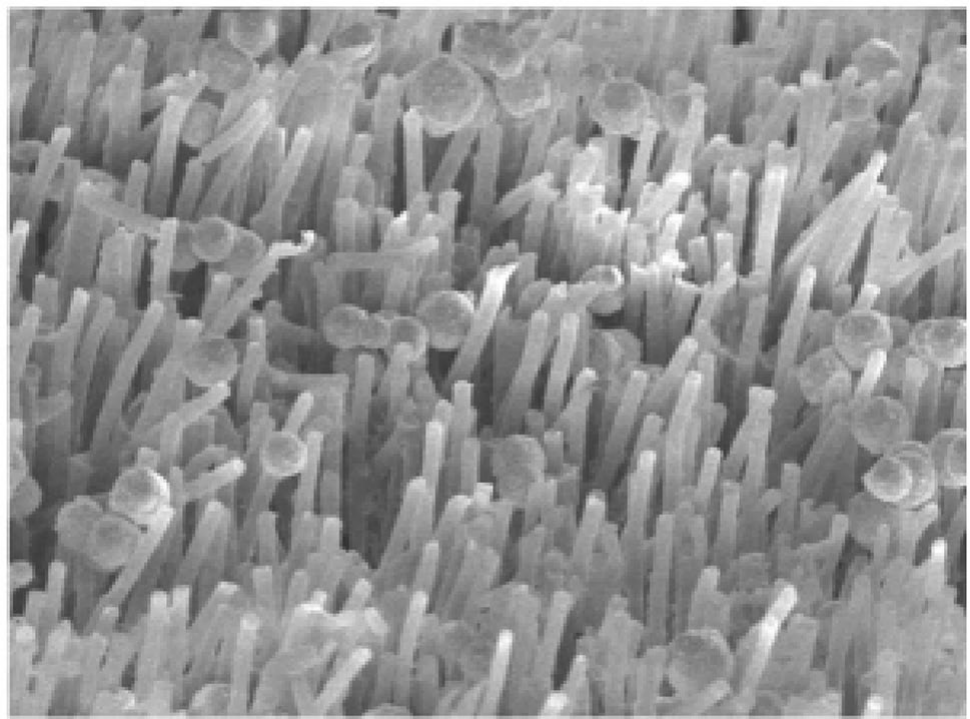
Example of a “gecko” type of nanostructure (fiber length 18 µm, diameter 0.6 µm)

Another very interesting approach with great potential for use in the design of gripping elements and heads is the application of materials with a high degree of surface adhesion. Currently, there are several technical solutions that are inspired by natural fibrous systems forming the contact surfaces of the limbs of small insects, spiders, and geckos. Their functioning has been studied for a relatively long time within the research programs of many universities and research institutes, and the mechanics of their functioning are described by various mathematical models depending on the boundary conditions and the structural geometry of the contact. A detailed comparison of the structures of organic fibrous surfaces together with a description of the adhesion mechanism are given in [15], moreover, in [16] the author presents an overview of SMART materials with externally stimulated adhesion as an interesting field of basic research. Based on targeted research, so-called GSA materials are now available [17–19], which are inspired by the biological gecko effect [20], whose surface is formed by a dense network of nanofibers [21] as seen in Fig. 5.

There are also adhesive materials with a specially structured surface [22, 23] or with surface treatments on a nanotechnology platform [24–27]. In general, these materials are divided into several basic groups characterized by hard polymers, soft polymers and nanomaterials using, for example, a surface carbon nanotube system. One of the many possibilities is the application of so-called “nano-pads” mainly represented by PU gels in various modifications with special treatment and an extremely adhesive surface [28]. The advantages of this material are that due to its patented modification it leaves no trace on the object after contact, and it is sufficiently resistant to mechanical damage. Therefore, use of its adhesive properties in the design of new types of gripping elements is of great interest.

To get an idea, following chapter presents a basic description of general adhesion mechanism of materials with fibrous surface structure. This is also widened by microscopic surface analysis of alternative material (PU gel) with a great potential of industrial application. *Chap. 2* describes the experiment verifying a PU gel function under various conditions of real operation. Results of the experiment were compared to theoretical background of mathematical description of adhesion contact presented in *chap. 3* and were subsequently used in numerical simulation of adhesive contact in *chap. 4*. Obtained knowledge finally allowed to prepare a new design solution of gripping element, presented in *chap. 5*.

### Materials with fibrous surface structure

The functioning of “gecko” adhesive gripping elements with characteristic surface fibres is based on the principles of chemical bonds, or interactions between molecules whose level is characterized by Van der Waals forces. The whole issue is relatively complicated, and according to available sources, three basic mechanisms contribute to the attraction of particles [29]. The first assumes that the potential energy *E*_*0*_ of the mutual orientation mechanism of action between two particles creates attractive binding forces, and is given by the equation

1 $$E_{0} = \frac{{2 \cdot p^{4} }}{{6\pi \varepsilon_{0} k_{B} T_{ABS} r_{M}^{6} }} ,$$

where *p* is the permanent dipole moment, *ε*_*0*_ is the vacuum permittivity (*8.82.10*^*–12*^* F.m*^*−1*^), *k*_*B*_ is the Boltzmann constant (*1.38.10*^*−23*^* J.K*^*−1*^), *T*_*ABS*_ is the absolute temperature, and *r*_*M*_ is the distance between the molecules.

The second mechanism is based on the contact of a dipole particle and a neutral particle, in which a dipole is subsequently induced. Both dipoles (permanent and induced) are attracted, and the interaction energy of the particles *E*_*i*_, depending on the permanent dipole moment of one particle, the polarizability of the other particle and their distance, is given by

2 $$E_{i} = \frac{{\alpha_{M} \cdot p^{2} }}{{2\pi \varepsilon_{0} r_{M}^{6} }} .$$

The last mechanism defines the interaction of two neutral particles without permanent dipole moments. The principle of the interaction in this case is the phenomenon in which the electrons in the electron shells of the particles are repelled, while at the same time the electrons of one particle get as close as possible to the nucleus, and the electrons of the other particle are on the opposite side of the nucleus. Previously neutral particles then become particles with dipoles creating attractive forces, whose energy *E*_*d*_ is defined so that

3 $$E_{d} = \frac{{3 \cdot \alpha_{M}^{2} \cdot E_{a} }}{{16\pi \varepsilon_{0} r_{M}^{6} }} ,$$

where *α*_*M*_ is the polarizability of the molecule and *E*_*a*_ is the characteristic of the particles lying numerically between the lowest excitation and the ionization energies of the particles. The total energy of the interaction of the particles is given by the sum of these energies, so that

4 $$E_{W} = E_{0} + E_{i} + E_{d} .$$

**Fig. 6:**
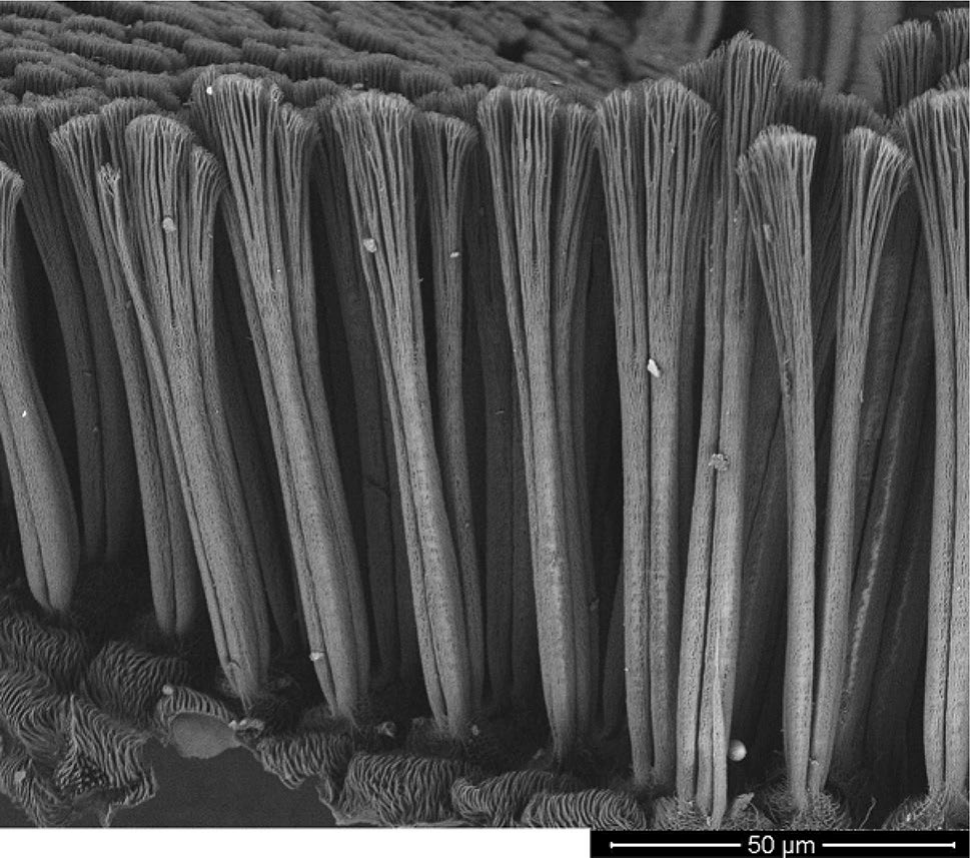
Microscopic image of gecko fibers (individual setae, incl. the spatulae at the ends) [32]

The total energy is proportional to the distance between the *r*_*M*_^−6^ molecules, and the acting forces are short ranged with the action in the phase where the particles are very close. The principle of copying the limbs of the gecko is applied the most in real applications [30, 31]. These are covered with fine keratin fibers (Fig. 6), known as setae with a length of approximately 30 to 150 µm and a density of approximately 5,000 fibers per mm^2^ (diameter of 100 to 200 nm). The individual fibers are further divided at the ends into another up to 1,000 formations, known as spatulae, which make contact with the microscopic irregularities of the surface and create attractive binding forces. Due to the effective transfer of this principle into practice, current efforts are focusing on the production of special surfaces from nanofibrous structures, or synthetic hydrophobic hair with high tensile strength and a modulus of elasticity in the range of 1 to 15 GPa.

Methods based on wax matrices with a nano- or micro-surface structure are used for production, which is performed by micro disruption using a nano-tool. The formed surface is filled with a polymer (often silicone rubber), which peels off or unsticks after curing.

To ensure the function, in addition to the length *l* and the diameter *d* of the fibers, it is necessary to ensure a minimum distance between the fibers *Δ*_*GF*_ so that they are not affected by the adhesive forces *F*_*A*_ at the ends. Simplifying the situation by converting to the task of a single-sided embedded beam gives a minimum distance of

5 $$\Delta_{GF} \ge \frac{{128F_{A} l^{3} }}{{3E\pi d^{4} }},$$

as mentioned in [33, 34], where the mechanism of contact of the fiber with the surface of the contact body is also described in detail.

### Materials based on PU gels

**Fig. 7:**
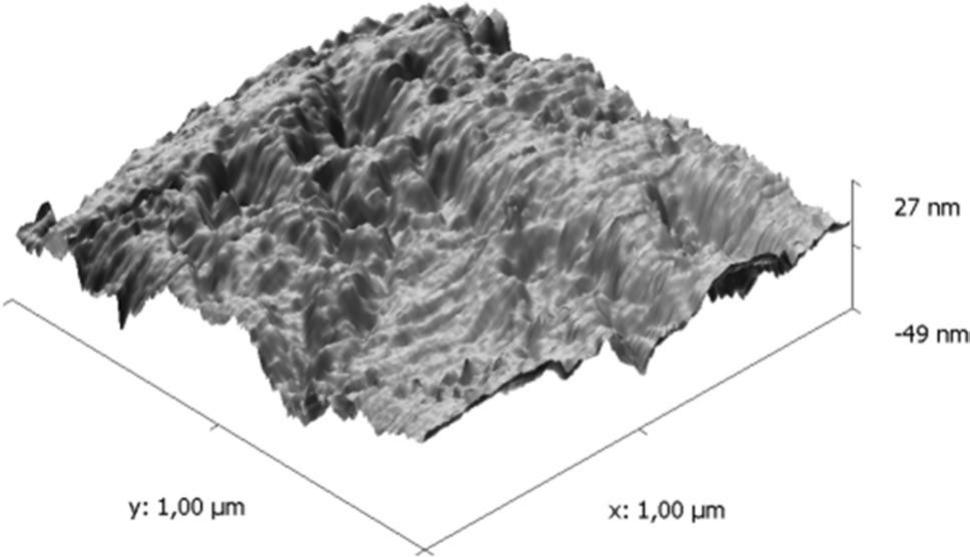
Surface structure of the magnified PU gel material

Due to the problematic mechanical resistance, complicated production technology, industrial availability and price of gecko materials, the authors’ attention focused on a PU gel material with a specifically structured surface, which was analyzed using both electron and confocal microscopes with a SW superstructure for three-dimensional imaging. A sample of the material was analyzed at different magnifications. The maximum possible magnification of the microscope approximately 60,000 times and the surface structure is shown in Fig. 7.

It is very difficult to generalize the findings of the microscopic analysis, but it is possible to state that no “hairs”, or “fibers” were found, the structure was mostly smooth to extremely smooth with irregular features in the interval from the minimum to the maximum in the range of *80 nm*. At the same time, manifestations of a disordered structure with a distinctive surface distribution were characteristic. In this case, it is not typical fibrous surface structure and adhesion energy will rely on surface energies and material compatibility, which is in detail discussed in *chap. 3*.

## Experimental verification of the functioning of the PU pads

**Fig. 8:**
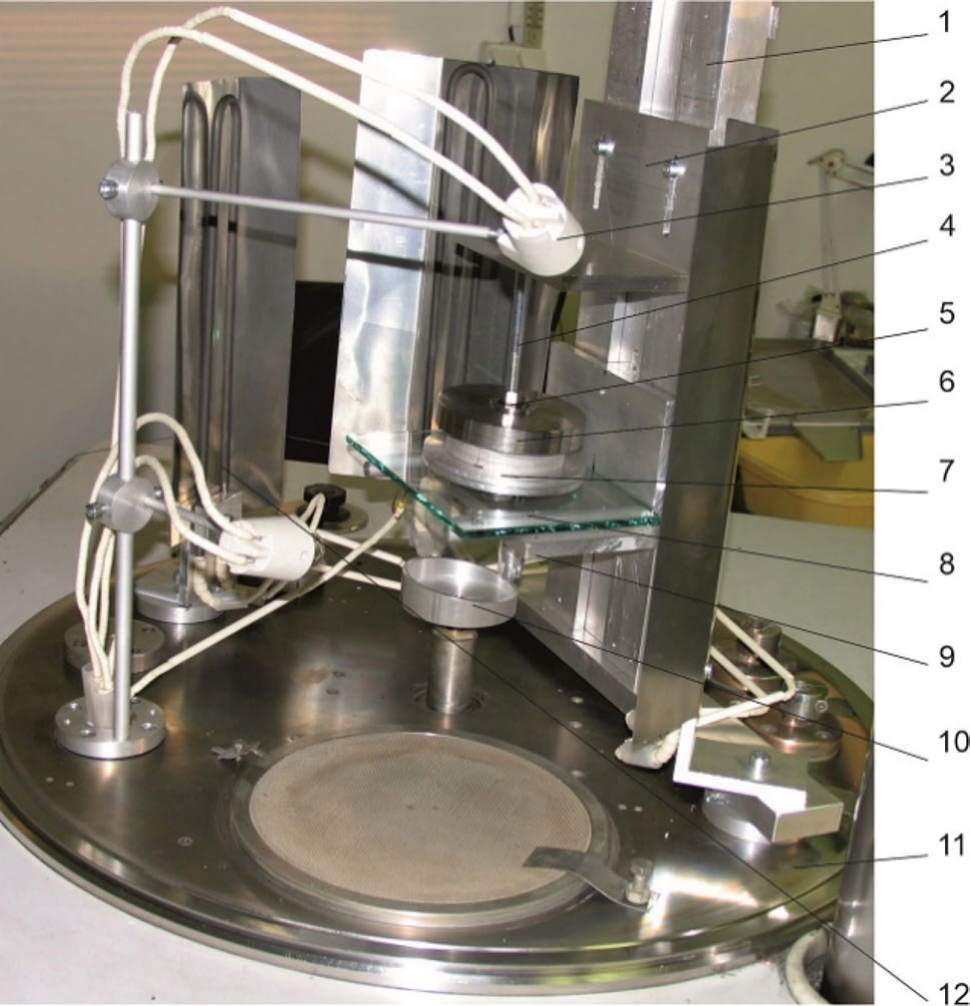
Experimental device (1—Frame of the device, 2—Fixed ramp, 3—Light, 4—Element hinge, 5—Ball joint, 6—Weight, 7—Gripping element, 8—Glass, 9—Movable support, 10—Cam, 11—Base of the vacuum chamber, 12—Resistance heating rods)

To verify the functional properties of the PU adhesive pads during the assumed manipulation tasks in production plants, as well as e.g., service robotics, a laboratory experimental device installable inside a vacuum chamber was assembled (Fig. 8). The designed device was used to perform an experimental analysis of the gripping process of the elements using the principle of adhesion under various operating conditions defined by the external load, ambient pressure, and temperature.

In detail, when experiment conditions are reached, firstly the gripping element *7* covered by the contact layer of PU gel with contacts the glass *8* of defined weight. In the second phase movable support *9* is released and the glass is held in stable position only by adhesion forces. This state causes external load of contact layer (PU gel) during which it is possible to analyse continuous changes in a contact with regard to time, displacement, initial load, etc. Obtained results are for needs of presented research and development discussed below.

### Testing of the functioning of the adhesive gripping principle in a vacuum

The reason for testing the adhesive element in a vacuum stems from the requirement to produce completely new products, which must be manipulated in vacuum chambers.

This production uses sophisticated technologies for applying specific functional layers, coatings, as well as special cleaning and decontamination processes.

When the technology is used in a vacuum, it is not possible to apply standard vacuum systems with suction cups, and this is an area where the application of new adhesive materials has a great potential. An interesting way of applying new materials with a high degree of surface adhesion is in the design of passive, or combined gripping elements operated either individually or in parallel depending on the geometrical characteristics and the weight of the object to be manipulated.

The targeted tests in a vacuum chamber under high vacuum conditions (0.005 Pa) succeeded in describing the relation between the pressure and the active surface of the element involved in the exertion of the gripping force. The gripping time of the held object with a defined weight was also analyzed in the assumed, mostly “quiet” manipulation mode, for which the passive gripping elements were primarily designed.

**Fig. 9:**
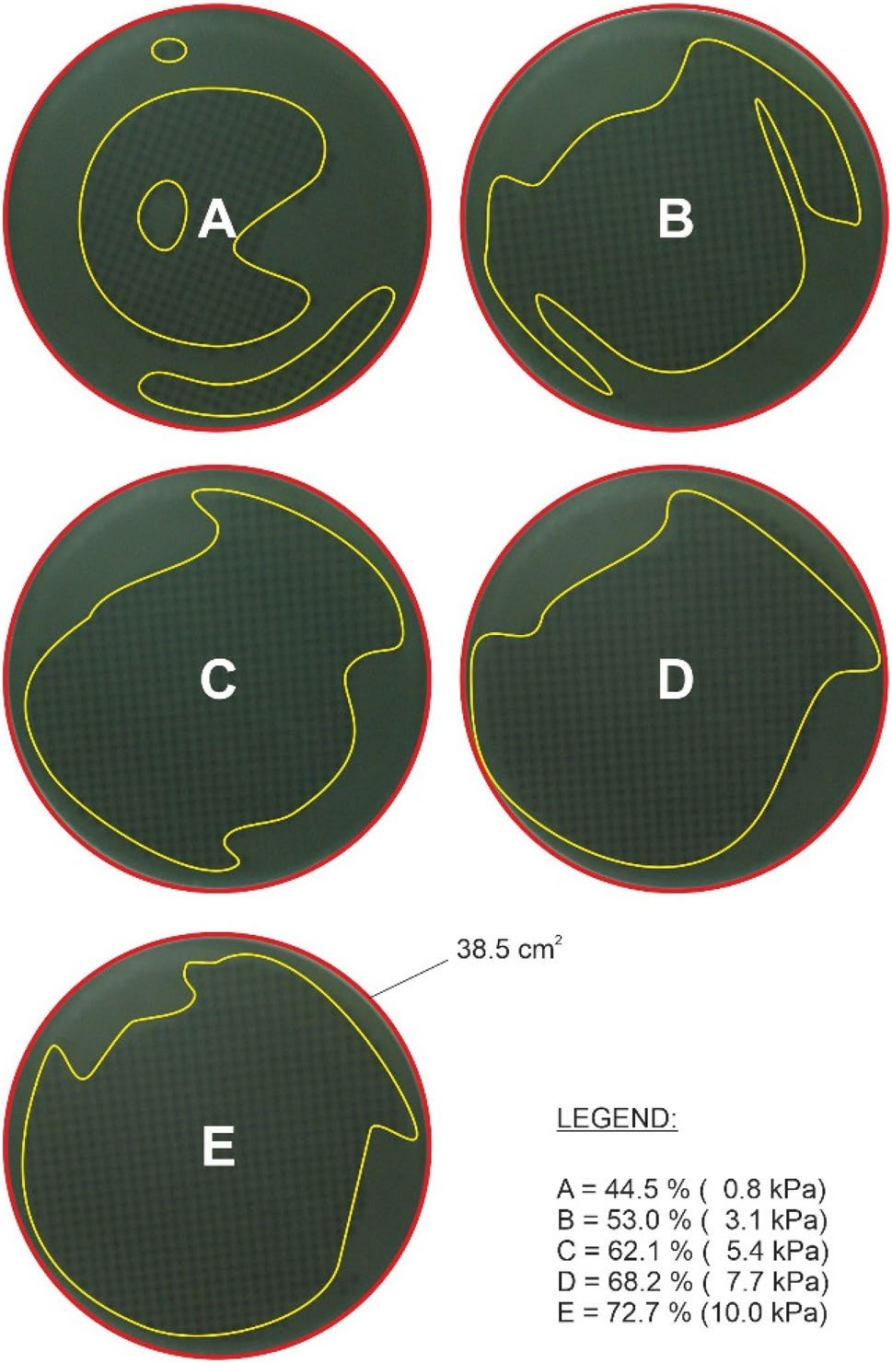
Active contact area of the element depending on the applied pressure (the data in the brackets)

From the acquired images of the contact quality (Fig. 9), it is possible to see that the contact area fluctuates significantly and ranges between 40 and 70% depending on the selected pressure, structure, and parallelism of the contact surface. The performed tests did not determine any significant effect of pressure (vacuum, atmospheric pressure) on the contact. On the contrary, the ambient temperature effected the rheological behavior of the adhesive pad, indirectly reducing the gripping time. However, it is evident that the nature of the contact had an overriding effect on the overall gripping force, contact stability, and gripping time, which are parameters that may fluctuate, and it is therefore necessary to consider a higher safety factor in the design phase.

**Fig. 10:**
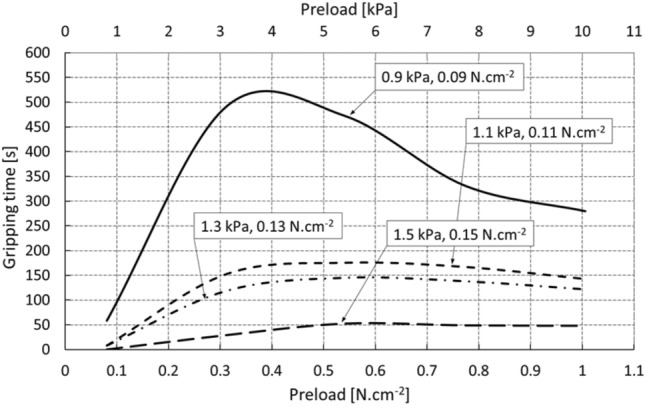
Gripping time of the object depending on the degree of pressure and load of the gripping element (the data in the boxes are for each curve)

Figure 10 shows that the optimal pressure with a time of action on the glass (the manipulated object) of around 10 s is in the interval of approximately 3 to 5 kPa, or 0.3–0.5 N.cm^−2^. It is clear that a significant increase in the force of the pressure leads to an increase in the size of the active area of the element (Fig. 9), but paradoxically not to a higher degree of adhesion or prolongation of the resulting gripping time due to the rheological properties of the tested material.

**Fig. 11:**
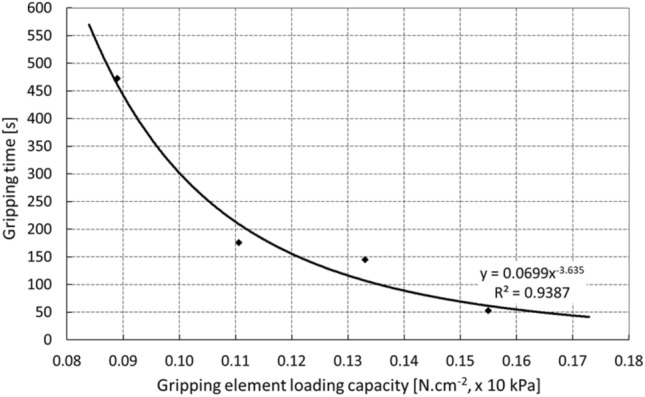
Gripping time of the object at the optimal initial element pressure

The graph in Fig. 11 shows the relationship between the loading capacity of the gripping element and the time of the adhesive gripping of the object at the optimum pressure and operating temperature of 25 °C. It is possible to state that the selected manipulation time (e.g., 150 s) corresponds to the loading capacity of the element 0.12 N.cm^−2^, which represents the limit equilibrium.

The introduction of a suitable degree of safety in the range of k = 3–5 may be considered a real basis for dimensioning the gripping element. It is evident that the gripping time logically affects the character of the contact forces, which may be monitored theoretically and predicted by computer modeling (*see Chap. 4*). Several approaches and techniques may be used to describe the system at the contact interface, where gradual detachment from the manipulated object occurs due to external loads, the boundary conditions, and the time-dependent rheological behavior of the adhesive layer. The Virtual Crack Closure Technique (VCCT) is based on the principle of fracture mechanics. The Cohesive Zone Model (CZM) uses a special approach in which the properties of elements are defined from the point of view of cohesive energy in the contact zones or the course of stress depending on profile displacement (traction), and stress criteria are used in Breaking Glued Contact (BGC) models of contact tasks [35, 36].

**Fig. 12:**
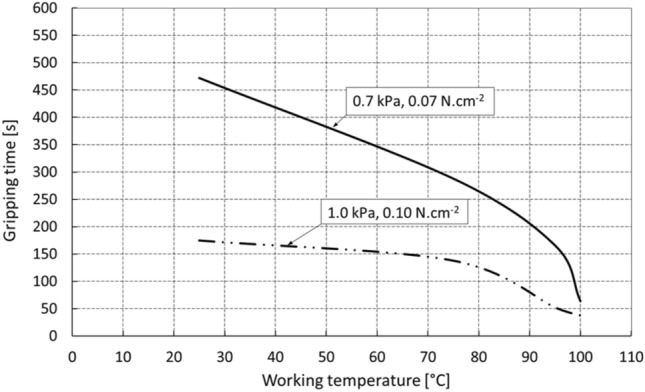
Gripping time depending on the operating temperature for different gripping element loads

The graph in Fig. 12 presents the dependence of the gripping time on the working temperature in the interval from 25 to 100 °C under conditions of the optimal pressure of the element of 0.4 N.cm^−2^ for 10 s when approaching the glass*.*

For a gripping element loaded with a pressure of 0.1 N.cm^−2^, the change in gripping time in the range of temperatures from 25 to 90 °C is from the initial 175 to 79 s, which represents a decrease of approximately 55%. Similarly, for a gripping element loaded with a pressure of 0.07 N.cm^−2^, the gripping time in the temperature range of 25 to 90 °C decreases from the initial 472 to 205 s, which represents a change in gripping time similar to the above of approximately 56%. In the region above 90 °C, the relaxation behavior of the nanomaterial is more pronounced and has not been fully described by previous experiments. Nevertheless, it is possible to state that the real designed system will be used as a gripping element in the design of modular gripping heads operating under high vacuum operating conditions at elevated temperatures of up to 100 °C, with the system providing a significant time reserve compared to the expected handling cycle time of approximately 30 s. It has been shown that the contact time changes significantly over time, especially in the first stages at temperatures of 80 and 90 °C, when it decreases abruptly to approximately half of the original values.

### Functioning of the adhesive material under atmospheric pressure conditions

The possibility to apply gripping elements using adhesive materials in passive modes of operation, i.e., without the need for an energy source, was demonstrated by the authors by performing extensive experiments at atmospheric pressure and temperatures of around 20 °C.

The dependence between the gripping time (contact) and the variable external axial load under the conditions of the static nature of the load was monitored. The experiments showed that due to its significantly rheological behavior, the adhesive material is mostly sensitive to the relaxation time, i.e., the delay between load cycles, which due to the optimal adhesion function is maintained at the level of tens of seconds, after which the surface layers fully relax. Another important factor to ensure the proper function of the material is the intensity of the initial pressure of the gripping element on the manipulated object, which was set for the tested material to a value of approximately 0.3 N.cm^−2^.

A relatively significant limitation in the use of adhesive materials is the state of the contact interface between the element and the manipulated object both in terms of the geometry (it is necessary to ensure parallelism) and the cleanliness of the contacting surfaces.

**Fig. 13:**
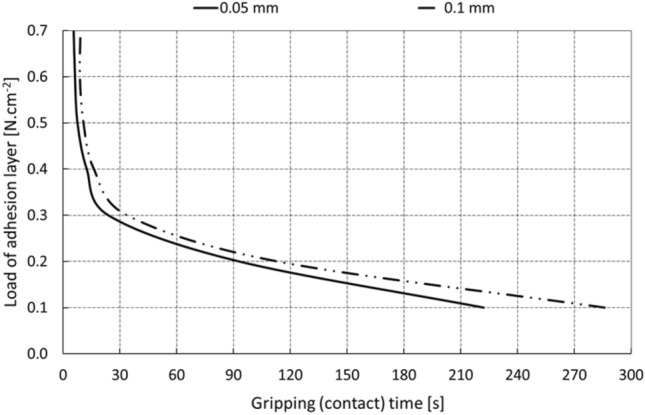
Contact (gripping) time for the selected level of contact profile displacement

The graph in Fig. 13 shows the relationship between the load of the adhesive layer and the contact time for preselected maximum levels of axial deformations of the layer of 0.05 and 0.1 mm.

**Fig. 14:**
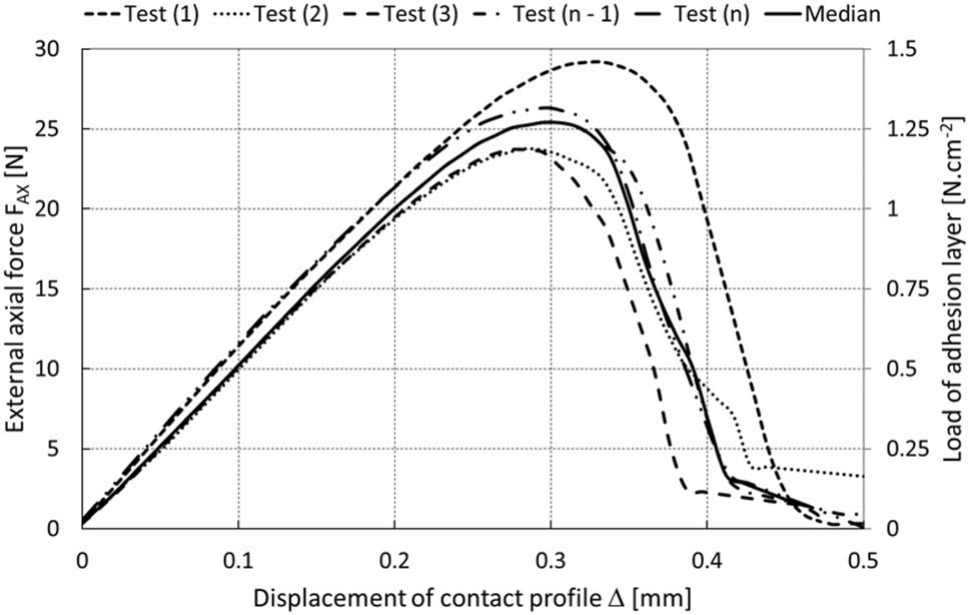
“Pull to break” test of the adhesive layer

**Fig. 15:**
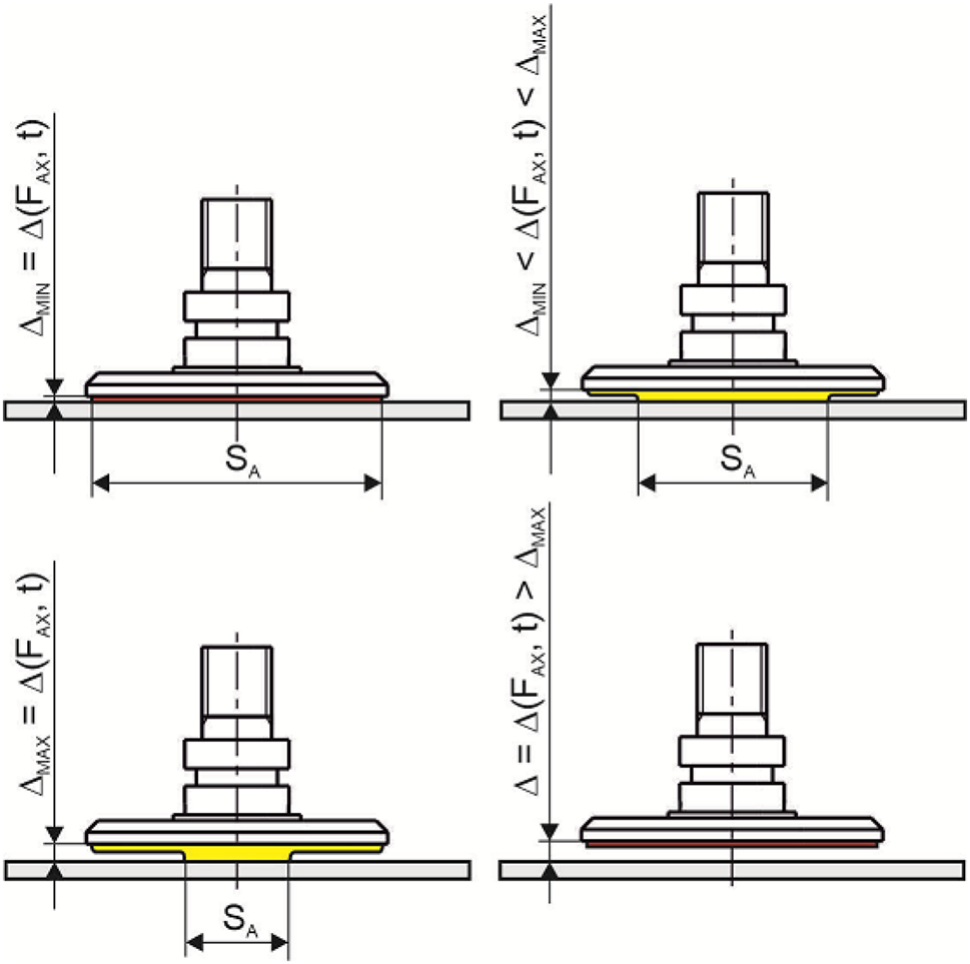
Presumed mechanism for tearing off the adhesive layer

The function of the layer was also monitored in relation to a continuously increasing axial load *F*_*AX*_ in time *t*, when the effective area *S*_*A*_ was decreased by tearing off the edges of the layer (or the center), the level of displacement *Δ* of the contact profile was increased, and there was a gradual transition from a stable to an unstable contact, which subsequently released the object (it collapsed), as presented in Figs. 14 and 15.

## Theory of the adhesive contact

From a theoretical point of view, the interaction of contacting bodies represents a relatively complex issue, where the resulting character and stability of the contact are affected by several parameters. These include the geometry of the contact surface, the rheological properties of the contact material, the condition of the surface in terms of contamination, chemical compatibility, and the crystallographic orientation of the surface, temperature, level, and distribution of contact forces (contact pressure), etc. Interesting is also a dry adhesion contact review with an overview of basic testing techniques presented e.g., in [37].

**Fig. 16:**
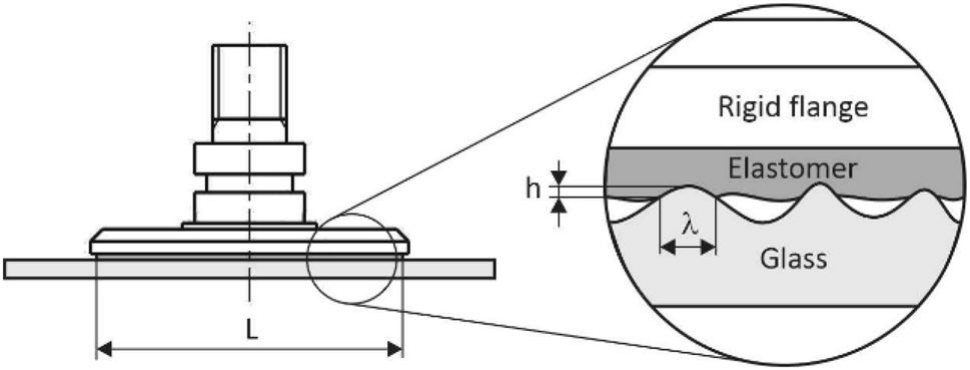
Microscopic contact profile of an elastomer with a relatively smooth glass surface

The current theory of contact mechanics based on the theory given in e.g., [38–40] assumes that the theoretical contact area may be described, as shown in *Fig. **16**,* by the function *A(λ)*, which defines the actual contact area depending on the geometry of the contact surface, or the distribution of the contact elements with a theoretical width *λ*.

The contact quality is then given by the ratio of the actual contact area and the theoretical (macroscopic) contact area as *P(ζ*_*CQ*_*)* = *A(λ)/A(L), whereby λ* = *L / ζ*_*CQ*_ and the contact quality factor is *ζ*_*CQ*_ ≥ *1*. Considering the mechanical properties of the contacting materials and in the absence of an additional external load, the quality of the contact between the elastomer (gripping element) and the glass (manipulated object) may be simply expressed by the ratio of elastic *U*_*el*_ and adhesive energy *U*_*ad*_ in the form

6 $$\theta = \frac{{U_{el} }}{{U_{ad} }} \approx \frac{{E \cdot \lambda \cdot h^{2} }}{{ - \Delta \gamma \cdot \lambda^{2} }} \approx - \frac{{E \cdot h^{2} }}{\Delta \gamma \cdot \lambda } ,$$

where *E* is the modulus of elasticity of the elastomer, *h* is the surface roughness, *λ* is the contact width and *Δγ* is the change in free energy per unit area in which the gripping element is in contact with the manipulated object, and the surface irregularities are displaced due to the rheological behavior of the material. A detailed mathematical analysis is given in e.g., [41, 42]. If it is applied that *U*_*el*_ = *-U*_*ad*_, i.e.,$h/\lambda \approx \sqrt{\Delta \gamma /E\lambda }$, then for very rough surfaces with a ratio *h/λ* = 1 and typical values *E* = 1 MPa and *Δγ* = 3 meV/Å^2^ (for rubbers), due to adhesion, the elastomer will be able to fill surface cavities *λ* < 0.1 μm. Conversely, for very smooth surfaces with *h/λ* = 0.01, the elastomer will be able to copy the rigid contact profile with the characteristic dimension *λ* = 1 mm.

In the case of a contact in which the contacting bodies are compressed by force *F*_*p*_, it will be necessary to subsequently apply force *F*_*a*_ characterizing the level of adhesion to redistribute them. The ratio between adhesion force *F*_*a*_ and force *F*_*p*_ is expressed by the adhesion coefficient, and the following is valid

7 $$f_{a} = \frac{{F_{a} }}{{F_{p} }} .$$

From a mathematical point of view, it is possible to define the adhesive energy [43] between contacting bodies with surface energies *γ*_*1*_ and *γ*_*2*_ and interfacial energy *γ*_*12*_ so that

8 $$U_{ad} = \Delta \gamma = \gamma_{1} + \gamma_{2} - \gamma_{12} .$$

Due to the fact that the interfacial energy *γ*_*12*_ is usually relatively small and difficult to measure, a material compatibility parameter *c*_*m*_ is introduced, which qualitatively characterizes the adhesive energy and may be written as

9 $$U_{ad} = c_{m} \cdot \left( {\gamma_{1} + \gamma_{2} } \right).$$

For identical metallic and non-metallic contact bodies *c*_*m*_ = 1, for compatible metallic bodies *c*_*m*_ = 0.5, non-metallic *c*_*m*_ = 0.6, and for e.g., incompatible non-metallic materials *c*_*m*_ = 0.36. In contrast to the classical concept of a strictly point contact, the geometry of the contact is influenced when considering adhesion forces [44–46] and the real contact behavior of a pair of flexible materials with respect to adhesion is very often replaced by the Johnson-Kendall-Roberts (JKR) model [47, 48]. For contacts of rigid bodies with very low deformability, the Deryagin-Muller-Toporov (DMT) model may be used. This model describes the phenomenon in which the adhesive forces act in close proximity to the contact surface. Due to the focus of the article, only the JRK model is presented in a simplified way. The model is based on the assumption that the contact pressure *p*_*C*_ between the spherical bodies 1 and 2 is given by the sum of the pressures *p*_*0.1*_ and *p*_*0.2*_ causing a uniform displacement, and in accordance with Hertz’s theory the following is true

10 $$p_{C} = p_{0,1} \cdot \left( {1 - \frac{{r^{2} }}{{a_{CS}^{2} }}} \right)^{\frac{1}{2}} + p_{0,2} \cdot \left( {1 - \frac{{r^{2} }}{{a_{CS}^{2} }}} \right)^{{ - \frac{1}{2}}} ,$$

where *r* is the radius and *a*_*CS*_ is the radius of the contact surface on the radius *r*. Assuming the elastic behavior of the contact surface of the elastomer, the resulting displacement of the profile is given as

11 $$u_{z} = \frac{{\pi a_{CS} }}{{E^{*} }} \cdot \left[ {p_{0,1} + \frac{1}{2} \cdot p_{0,2} \cdot \left( {1 - \frac{{r^{2} }}{{2a_{CS}^{2} }}} \right)} \right],$$

or in relation to the penetration displacement δ as

12 $$u_{z} = \delta - \frac{{r^{2} }}{2R} .$$

Based on the above assumptions, it is possible to determine the contact pressures for which the following is true

13 $$p_{0,1} = \frac{{2a_{CS} E^{*} }}{\pi R} , \; p_{0,2} = \frac{{E^{*} }}{\pi } \cdot \left( {\frac{\delta }{{a_{CS} }} - \frac{{a_{CS} }}{R}} \right) = 2 \cdot \sqrt {\frac{{\Delta \gamma E^{*} }}{{\pi a_{CS} }}} ,$$

where

14 $$\frac{1}{{E^{*} }} = \frac{{1 - \mu_{1}^{2} }}{{E_{1} }} + \frac{{1 - \mu_{2}^{2} }}{{E_{2} }} {\text{ and}}\; \frac{1}{R} = \frac{1}{{R_{1} }} + \frac{1}{{R_{2} }},$$

provided that E^*^ is the contact modulus and *R* is the reduced (relative) contact radius. Subsequently, the total contact energy is characterized by the sum of the elastic and adhesive energies such that

15 $$U_{T} = U_{el } + U_{ad} = \frac{1}{2} {\iint }p_{C} \cdot u_{z} dxdy + \Delta \gamma \cdot \pi \cdot a_{CS}^{2} .$$

The displacement δ will further depend on the equilibrium radius of the contact surface, which will define the minimum of the total energy, i.e., $\partial {U}_{T}/\partial {a}_{CS} =0.$ With this assumption, the relationship between δ and *a*_*CS*_ may be defined by the relationship

16 $$\delta = \frac{{a_{CS}^{2} }}{R} \pm \sqrt {\frac{{2\Delta \gamma \pi a_{CS} }}{{E^{*} }}} .$$

In addition, for the adhesive force on a spherical surface, the following is valid

17 $$F = - \frac{{dU_{T} }}{d\delta } = - \frac{{\partial U_{T} }}{\partial \delta } = E^{*} \cdot \left[ {2\delta a_{CS} - \frac{2}{3}\frac{{a_{CS}^{3} }}{R}} \right] {\text{for}} \frac{{\partial U_{T} }}{{\partial a_{CS} }} = 0$$

and for the final value of the displacement δ the force will be

18 $$F = \frac{{4E^{*} a_{CS}^{3} }}{3R} - \sqrt {8\Delta \gamma \pi E^{*} a_{CS}^{3} } .$$

The force defined in relation (18) has a minimum for the critical contact radius for which the following relation applies

19 $$a_{c} = \left( {\frac{9}{8} \cdot \frac{{\Delta \gamma \pi R^{2} }}{{E^{*} }}} \right)^{\frac{1}{3}} .$$

With regard to the critical contact radius, it is possible to further simplify relationship (18) so that the minimum adhesive force that would have to be exceeded in the event of a break in contact (adhesion) will be

20 $$F_{\min } = - \frac{3}{2} \cdot \pi R\Delta \gamma .$$

For the theoretical analysis of the behavior of the adhesive contact of the gripping element and the glass as the manipulated object, the methodology of Cohesion Zone Modeling (CZM) was applied, which is very often used in engineering practice and is part of several computer software packages [49–51].

The methodology was introduced in the early 1960s, and is based on the description of the relative displacement of a pair of contact points at the contact interface as a function of traction, which in this case is the force per unit area.

The computational elements describing the cohesion zone do not include material properties, but characterize the cohesive tensile forces and displacements associated with a gradual collapse of the contact, e.g., during external loading of the contacting bodies, and there are several criteria by which the cohesion zone may be parameterized [52–54].

**Fig. 17:**
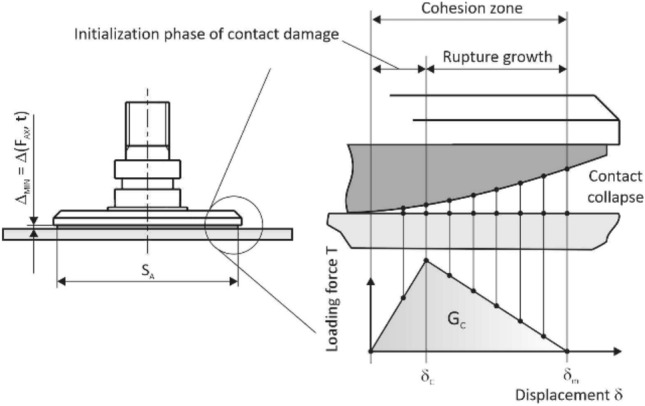
Gradual relative displacement of the contact at mutual points

**Fig. 18:**
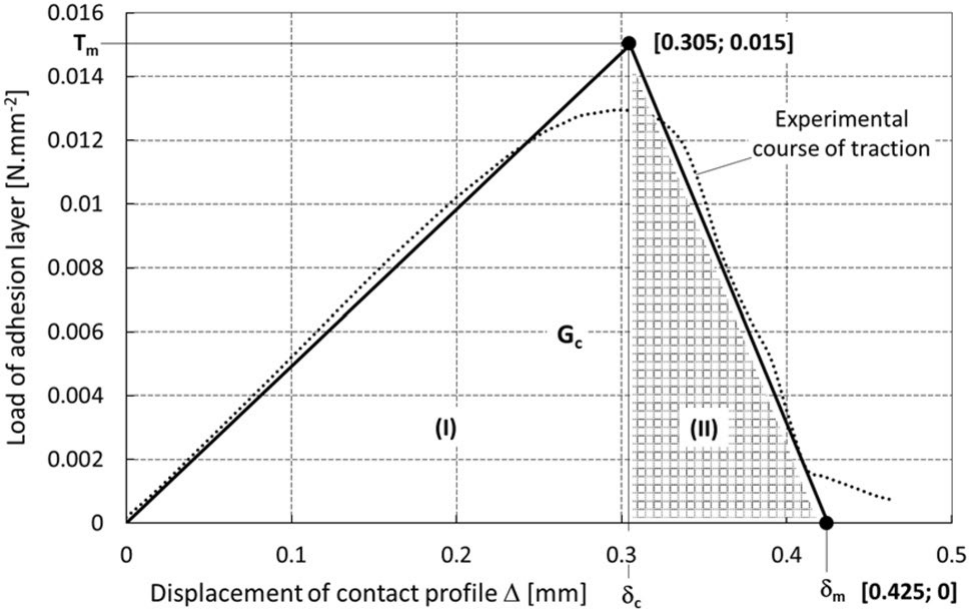
Bilinear contact model (zone I—reversible elastic behavior, zone II—irreversible plastic behavior)

In general, the relationship between the traction and displacement may be expressed linearly or non-linearly, with either a continuous or discontinuous character. The collapse of the contact (Fig. 17) is usually defined by the maximum traction *T*_*m*_, or critical displacement *δ*_*c*_, and subsequently the so-called critical energy, which is gradually released during loading. It was possible to determine the parameters of the cohesion zone based on the performed experimental measurements and define them in accordance with the graph in Fig. 18.

Two models may be used to describe the dependence of traction on displacement, i.e., a bilinear or exponential model. Due to the nature of the experimentally obtained data (Fig. 14), a bilinear model was used to determine the critical cohesive energy, where

21 $$\begin{aligned} G_{c} & = \frac{1}{2} \cdot T_{m} \cdot \delta_{c} + \frac{1}{2} \cdot T_{m} \cdot \left( {\delta_{m} - \delta_{c} } \right), \\ G_{c} & = \frac{1}{2} \cdot T_{m} \cdot \delta_{c} + \frac{1}{2} \cdot T_{m} \cdot \delta_{m} - \frac{1}{2} \cdot T_{m} \cdot \delta_{c} , \\ G_{c} & = \frac{1}{2} \cdot T_{m} \cdot \delta_{m} . \\ \end{aligned}$$

Specifically, *G*_*c*_ = 3.2 J.m^−2^, the critical displacement *δ*_*c*_ = 0.305 mm, and the maximum displacement *δ*_*m*_ = 0.425 mm. In addition, to ensure the optimal stability and convergence of the calculation, a viscous damping parameter with a viscous energy factor of 0.005 was introduced into the computer model, which corresponds to the relation

22 $$T_{v} = \min \left( {\frac{{f_{v} T_{m} \dot{\delta }}}{{\dot{\delta }_{r} }},T_{m} } \right),$$

where *T*_*v*_ is the viscous traction, $\dot{\delta }$ is the function of the equivalent displacement velocity, $\dot{{\delta }_{r}}$ is the reference value of the equivalent displacement velocity, and *f*_*v*_ is the viscous energy factor.

**Fig. 19:**
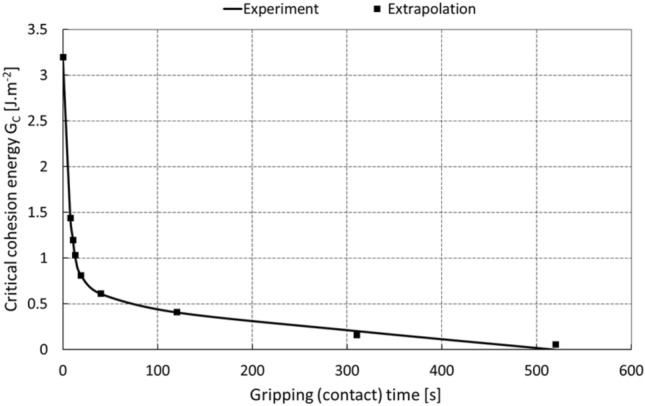
Change in critical cohesive energy over time

In addition to the fact that the critical cohesive energy is dependent on traction and displacement, it is also necessary to take into account other boundary conditions such as temperature, ambient pressure, and the duration of the external loading force. Generally, in the case of adhesive, mainly glued joints, it is assumed that the joint degrades in dependence on temperature, which was also demonstrated here for the potential case of using an adhesive gripping element at high temperatures, by the series of experiments in *Chap. 2*. Under normal manipulation conditions with no significant temperature changes, the dominant parameter influencing the quality and stability of the contact is the load time, when in the first tens of seconds of adhesive gripping there is a significant decrease in cohesive energy. The development of the contact quality over time is evident from the graph in Fig. 19, which summarizes the results of the experiments performed. For the needs of the computer modeling, the dependence of cohesion energy on time was extrapolated by a nonlinear regression curve in an exponential form by a four-parameter relation

23 $$G_{c} \left( t \right) = A \cdot e^{B \cdot t} + C \cdot e^{D \cdot t} ,$$

where *t* is the loading time of the constant force of the gripping element, and the parameters *A, B, C* and *D* are real numbers *(A* = 0.7416,* B* =  − 0.004894*, C* = 2.458, *D* =  − 0.1527).

## Computer simulation of the adhesive contact

**Fig. 20:**
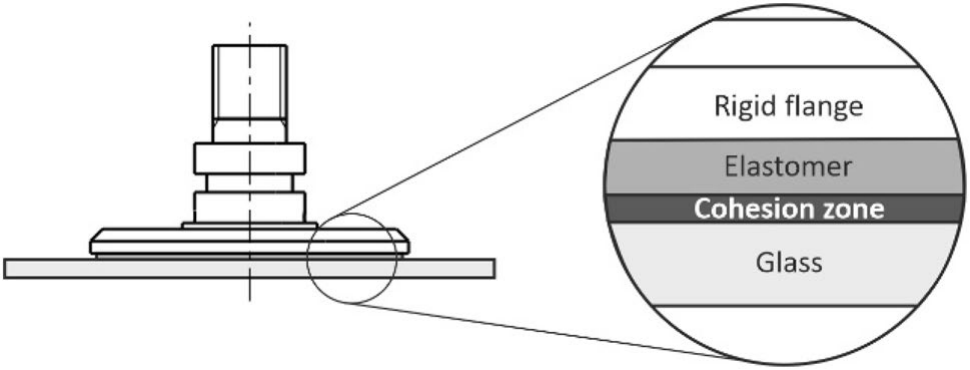
Composition of the computer model

Based on the analysis of the experimental data performed in *Chap. 2,* and the analysis of the theory of adhesive contact in *Chap. 3*, a computer model of the contact of the adhesive gripping element with a smooth glass surface was designed and assembled in the MSC.Marc software product (Fig. 20).

The real behavior of the polyurethane-based elastomer was replaced by the Mooney-Rivlin rheological model with material constants c_10_ = 139,352 Pa, c_01_ = 0 Pa, c_11_ = 0 Pa, c_20_ = 17,963 Pa, c_30_ = 4579 Pa, which were determined based on the results of laboratory tests on tear machines and are consistent with the relatively wide modulus of elasticity range from *0.00379 to 0.248 GPa* reported for PU-based synthetic elastomers. The glass (the manipulated object) was described by an elastic model with the modulus of elasticity *E* = 72.5 GPa, Poisson constant *ν* = 0.22, and density value *ρ* = 2,500 kg.m^−3^.

**Fig. 21:**
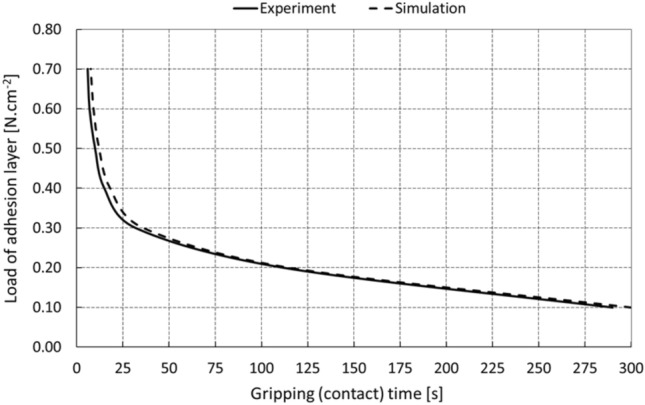
Course of the contact time

The results of the computer simulation showed a very good agreement in comparison with the experimental data. The measured differences were up to 3% and are evident from the course of the dependence of the contact time on the load level of the adhesive layer, i.e., the contact element, in the axial direction from the graph in Fig. 21.

**Fig. 22:**
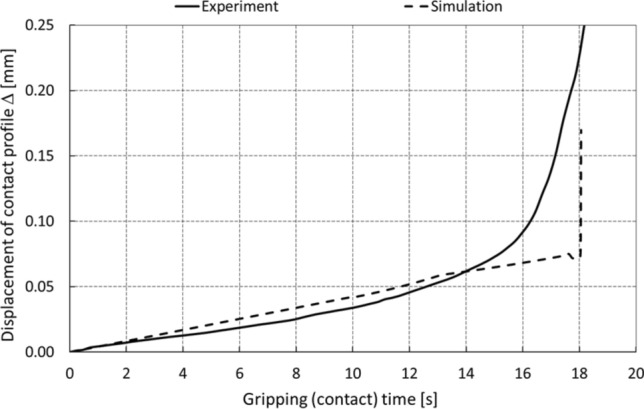
Development of the contact collapse

In general, it may be stated that the point of contact collapse based on the computer simulation, and in contrast to the experiments, is characterized by a step change, which is evident from the graph in Fig. 22 for a selected axial load at a level of e.g., 0.4 N.cm^−2^. Optimisation (refinement) of computing step allows minimizing the extreme point of collapse and thus coming closer with the computer simulation results to experimental course.

**Fig. 23:**
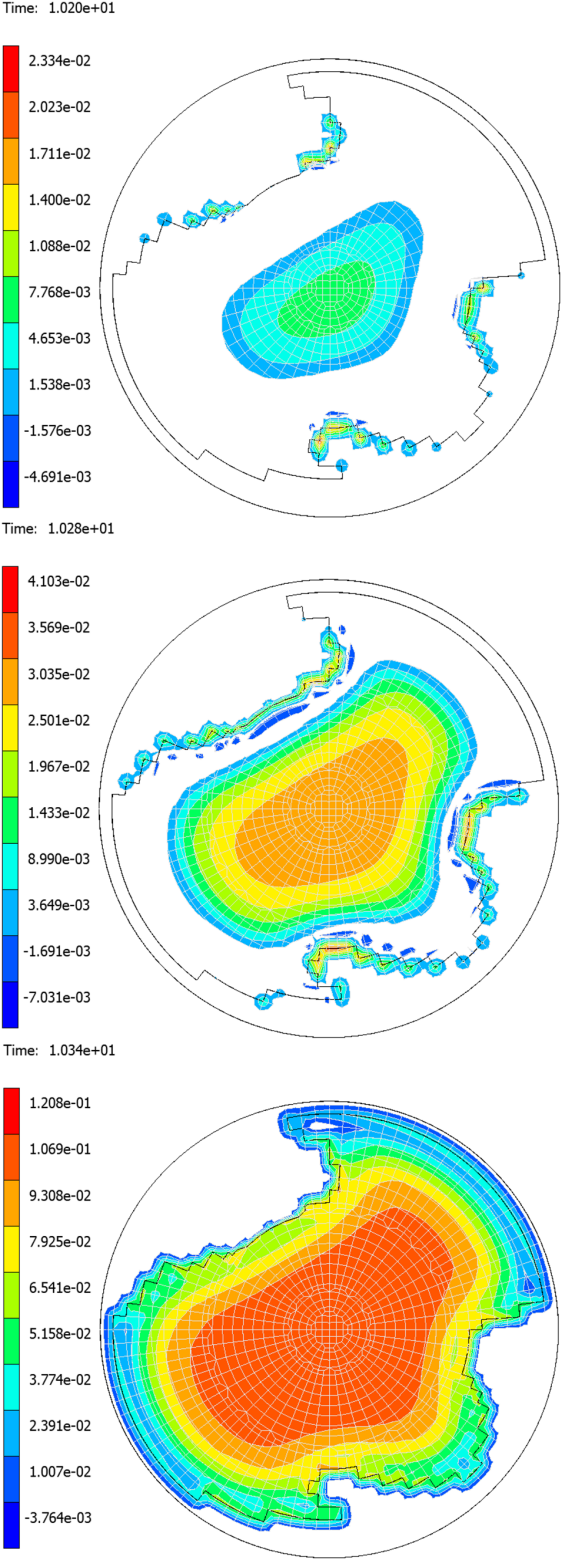
Development of the adhesive contact collapse

The representativeness of the computer model is also demonstrated in Fig. 23, which shows the gradual development of the collapse of the adhesive contact corresponding to the initial distribution of the “C” type contact surface described in *Chap. 2.1.*

## Practical use of the achieved results and discussion

Vacuum manipulation of objects is usually performed by means of vacuum gripping elements, known as suction cups, where the respective gripping force is derived by the difference between the pressure in the space between the suction cup and the object, and the ambient pressure (usually atmospheric pressure). It is recommended to load the suction cups in the axial direction, and the generally known calculation procedure during dimensioning corresponds to this. In other modes, in which the loads are radial with tilting and torque, or a combination of the two, the disadvantage is a significant decrease in the level of contact stability and safety of the manipulation of the object, leading to collapse due to the mechanical properties of the suction cups.

### Combined gripping elements

**Fig. 24:**
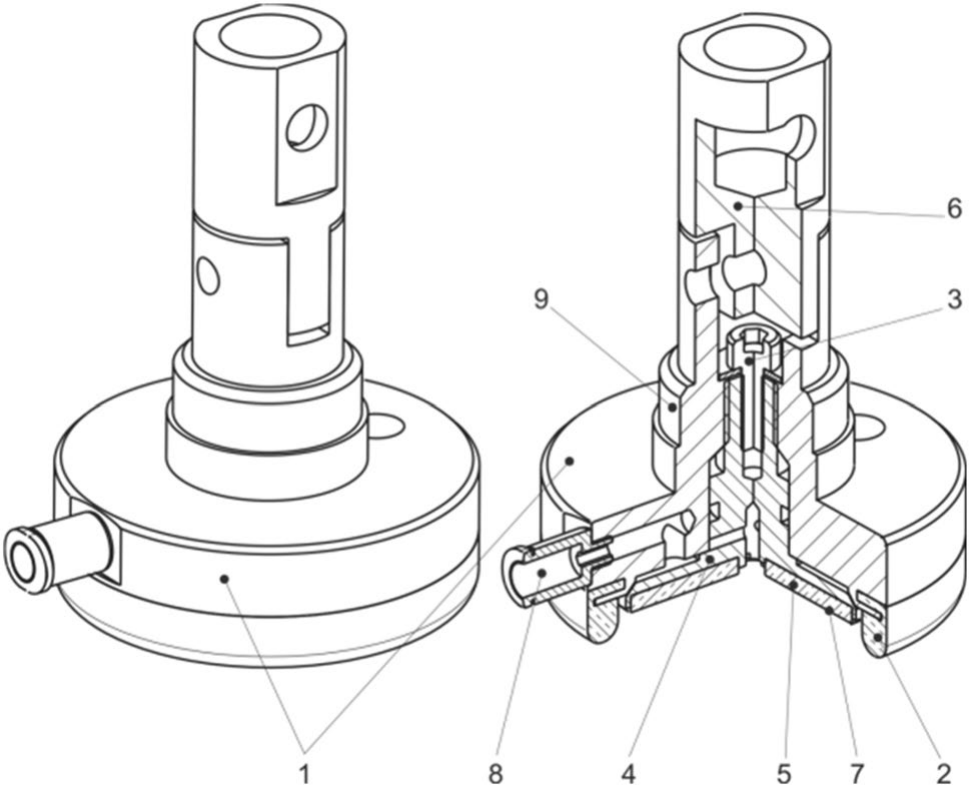
Vacuum adhesive gripping element

The technical solution [55], which is based on a combination of a vacuum gripping element with a rigid flange, a flexible sealing flange, and a retractable adjustable plate provided with an adhesive layer (Fig. 24), considerably compensates for the given shortcomings, and significantly increases the loading capacity of the vacuum gripping elements.

The designed element consists of the basic body of the element *1*, which is fitted around the circumference with a flexible sealing rim *2*, which, after contact with the manipulated object, forms a vacuum-tight cavity. In the cavity, it is possible to regulate the vacuum level by connecting a vacuum source through the opening *8*. A support plate 4 fitted with an adhesive layer *5* is inserted inside the element body and may be positioned together with the layer with respect to the contact plane by a threaded connection with a locking screw *3*. The plate 4 also forms a planar contact surface *7* with the layer *5*. The cylindrical surface with the thread *9* forms the connection interface.

**Fig. 25:**
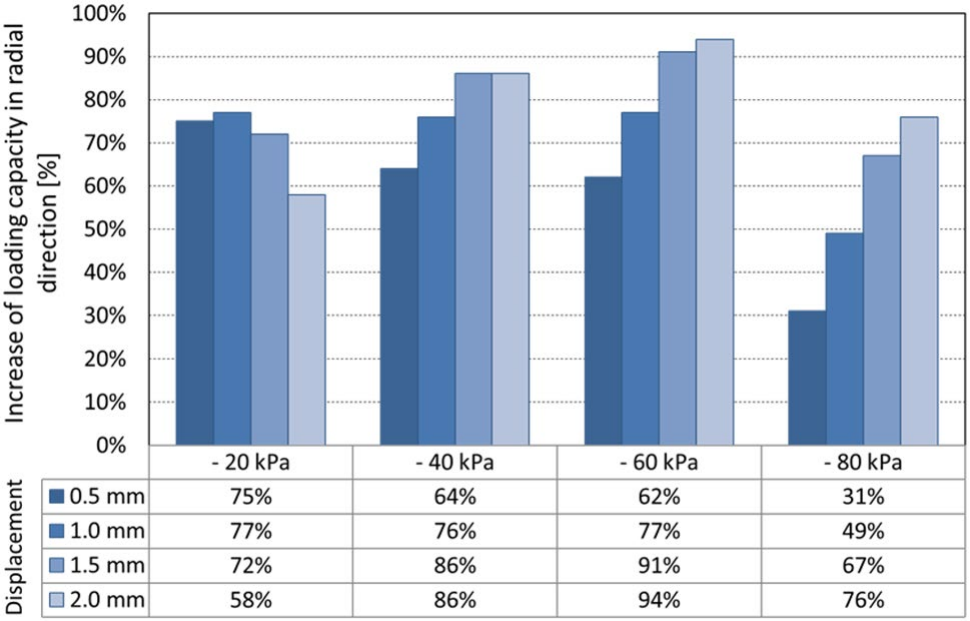
Increase in the loading capacity of the gripping element

The performed laboratory tests of the designed element clearly confirmed that the adhesive layer together with the support plate have a significant effect on the stability of the grip (contact), which is manifested by an effective increase in the loading capacity (Fig. 25) in the range of approximately 30 to 95%.

With a stable contact defined by the selected maximum displacement of 0.5 mm, there is an average increase in the loading capacity of *60%*. It has also been shown that a deviation of the position of the support plate in the order of tenths of a millimeter from the optimal position may lead to a reduction in the effect and the loading capacity of the element in the radial direction does not increase to the expected extent.

Quite different tendency characterized by gentle decrease of load regards to a profile displacement for a vacuum − 20 kPa is caused by relatively small gripping force, which creates only low pressure of adhesive layer. Using higher, in industry commonly used under-pressures eliminates this phenomenon.

It is evident that when the support plate is extended above the level of the sealing rim, it is possible to use the element without the need to evacuate the space between the element and the manipulated object and to operate the system in a passive mode without the need to connect an energy source.

The positioning of the support plate (adhesive layer) with the element body using a threaded connection is considerably inaccurate and insufficiently flexible, because for each geometry of the element it is necessary to set its optimal position, which is not known in advance and requires testing. Therefore, an optimized design solution was proposed, in which after the contact of the gripping element *1* with the manipulated object *7*, a closed space *10*, or *11* is created, and sealed by a sealing flange *2*. Subsequent evacuation of the air in the space through the opening *8* pushes the support plate 4 into an optimal contact with the object *7* by means of a piston *3*. An integral part of the plate 4 is the adhesive layer *5*, which forms the contact interface *12* together with the object *7*. The level of pressure between the plate 4 or the layer *5* and the object *7* may be regulated by the value of pressure in the regulation space *9* above the piston *3*, whereby optimal pressure levels can be achieved depending on the deformation of the sealing rim *2*, and the mechanical properties and surface profile of the object *7,* which ensures the maximum increase of friction forces during manipulation.

**Fig. 26:**
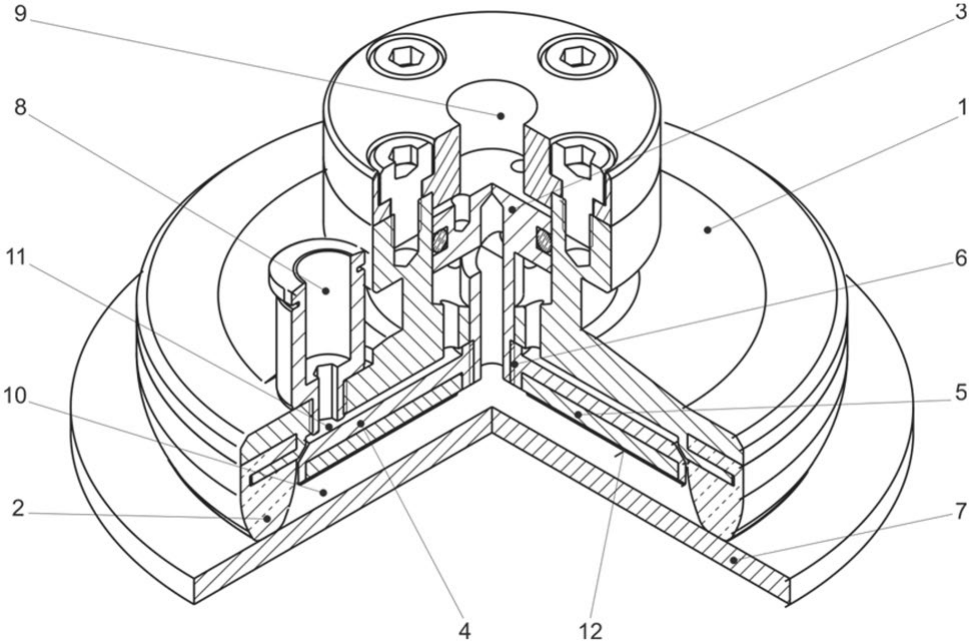
Gripping element (without supporting plate orientation compensation)

The basic technical design is shown in Fig. 26, in which the detailed cross-section shows the arrangement of the gripping element with a rigid threaded connection *6* of the support plate and the piston.

**Fig. 27:**
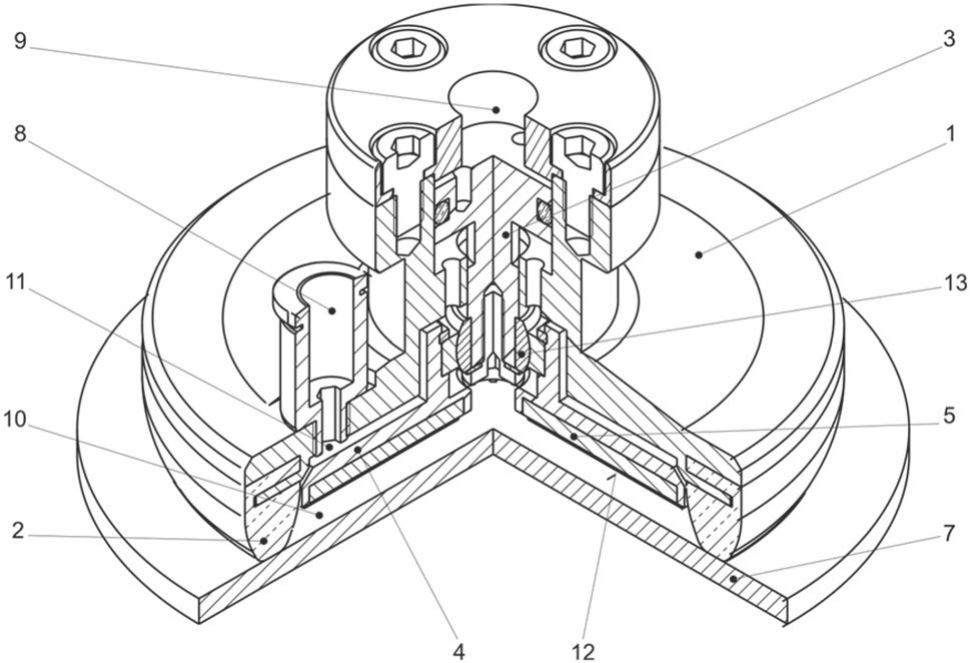
Gripping element (with support plate orientation compensation)

Figure 27 shows a solution enabling automatic adjustment (change) in the orientation of the adhesive layer, or the support plate, depending on the orientation of the contact surface of the object (the plate and the piston are connected using a ball joint *13*) in a position where the adhesive insert is out of contact with the manipulated object.

Based on the technical solution, the gripping element may be used in a wide range of manipulation and gripping processes with higher demands on the safety of the gripping and holding of the object. It is suitable for e.g., manipulating flexible objects (minimization of elastic deformations in objects with low transverse stiffness), and manipulation with a vertically oriented gripping plane, etc. It is also suitable for objects with rough and uneven surfaces, and generally for applications where external forces act parallel to the gripping plane. The solution also minimizes the consumption of compressed air, while maintaining a level of force response.

### Adhesive gripping element

**Fig. 28:**
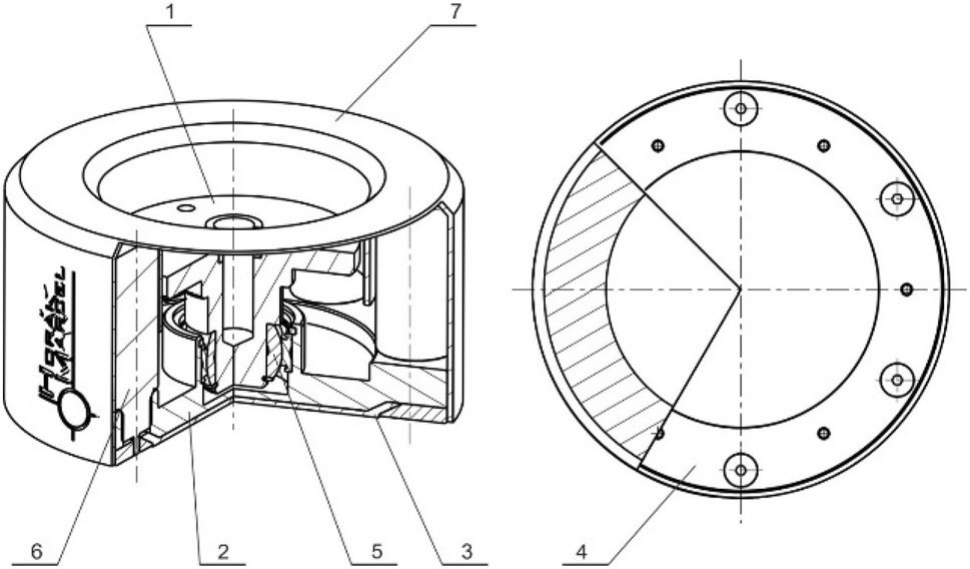
Adhesive gripping element with an electromagnetic drive

An interesting alternative to the combined gripping element is the compact solution of the passive gripping element (Fig. 28) with a cover *7* (bottom right), which consists of a duralumin flange *1* with an adjoining tilting plate 2 fitted with an adhesive layer *3*, which is circumferentially secured against detaching by a pressure retaining ring *4*, and at the same time is attached to the tilting plate. The tilting of the plate allows an articulated connection *5* with the flange *1*, ensuring the automatic orientation of the adhesive layer depending on the position of the contact plane of the manipulated object. Active interference of the gripping adhesive force is provided by a system of tubular electromagnets *6* circumferentially mounted in the body of the tilting plate.

Manipulation with flat objects is usually performed by means of multi-element gripping heads which form a frame fitted with gripping elements in a parallel arrangement. Positional inaccuracies depending on the geometry and mechanical properties of the head frame and the manipulated object in relation to the gripping plane are minimized by spring compensators and articulated mounting of the gripping element, and partly by the material characteristics of the gripping element. This system is sufficient for applications of active gripping elements with an adequate effective stroke.

In passive systems using adhesion to derive the gripping force, the problem is that when approaching and pushing the head onto the object in a detachable position the gripping plane is compensated, but further axial displacements occur during subsequent manipulation, due to flexible mounting of the elements and deformation of the object (especially for objects with low stiffness). These displacements are a source of uneven loading of the elements, and lead to a drastic reduction in safety, contact stability, and consequently to collapse of the grip.

**Fig. 29:**
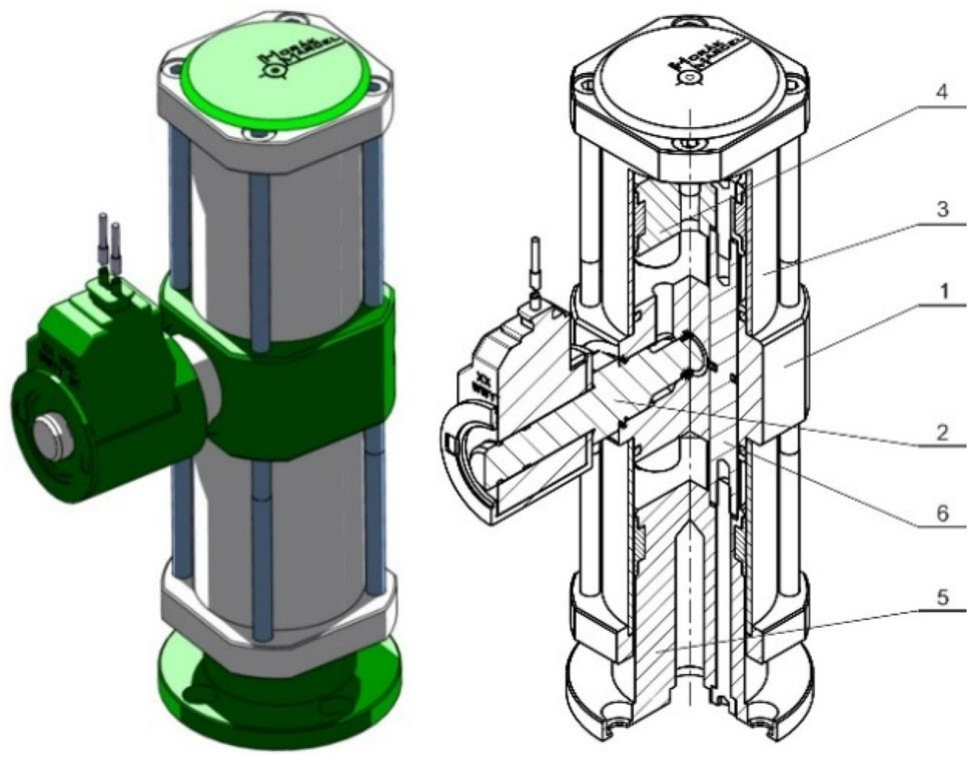
Fluid position compensator

Consequently, the concept of a fluid position compensator was proposed for the purpose of position compensation (Fig. 29). The idea is that a working fluid is contained in the divided space between a pair of firmly connected pistons *4* and *5* moving in the cavity of the cylinder *3*. The space between the pistons is separated by a connecting block *1* with a baffle in the flow channel actively controlled by a seat valve *2*. So as not to rotate relative to the connecting block, the pistons are mechanically connected to each other by a pair of piston rods *6* symmetrically located to the piston axis and provided with seals to prevent leakage of the fluid. By regulating the baffles in the channel of the connecting block, position compensation is achieved when the gripping element is in contact with the object, as well as position locking during the manipulation process, which achieves an optimal leveling of the contact plane and minimizes the effect of any uneven loading of the individual gripping elements.

**Fig. 30:**
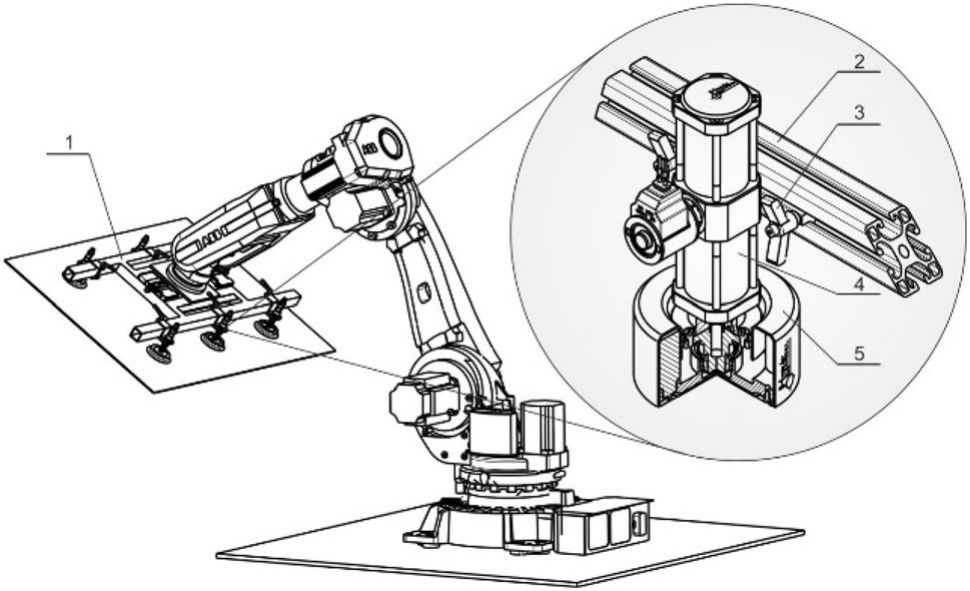
Industrial robot with multi-element gripping head

Figure 30 shows in detail the possibility of replacing the classic vacuum gripping element combined with spring position compensators and dynamic shocks with a new energy saving system applying a gripping element system according to the patent in [56] extended by an active adhesion force suppression system *5* and fluid position compensator *4* according to the utility model in [57] with a sliding lock *3* on the frame of the gripping head *2* or *1*.

In relation to the above, it should be noted that it is possible to use various simpler mechanical compensators to provide the position compensation, e.g., on a platform from Destaco or Schmalz, etc., which, however, do not allow for continuous automatic compensation, and which require the appropriate settings and corrections of the position to be made manually.

**Fig. 31:**
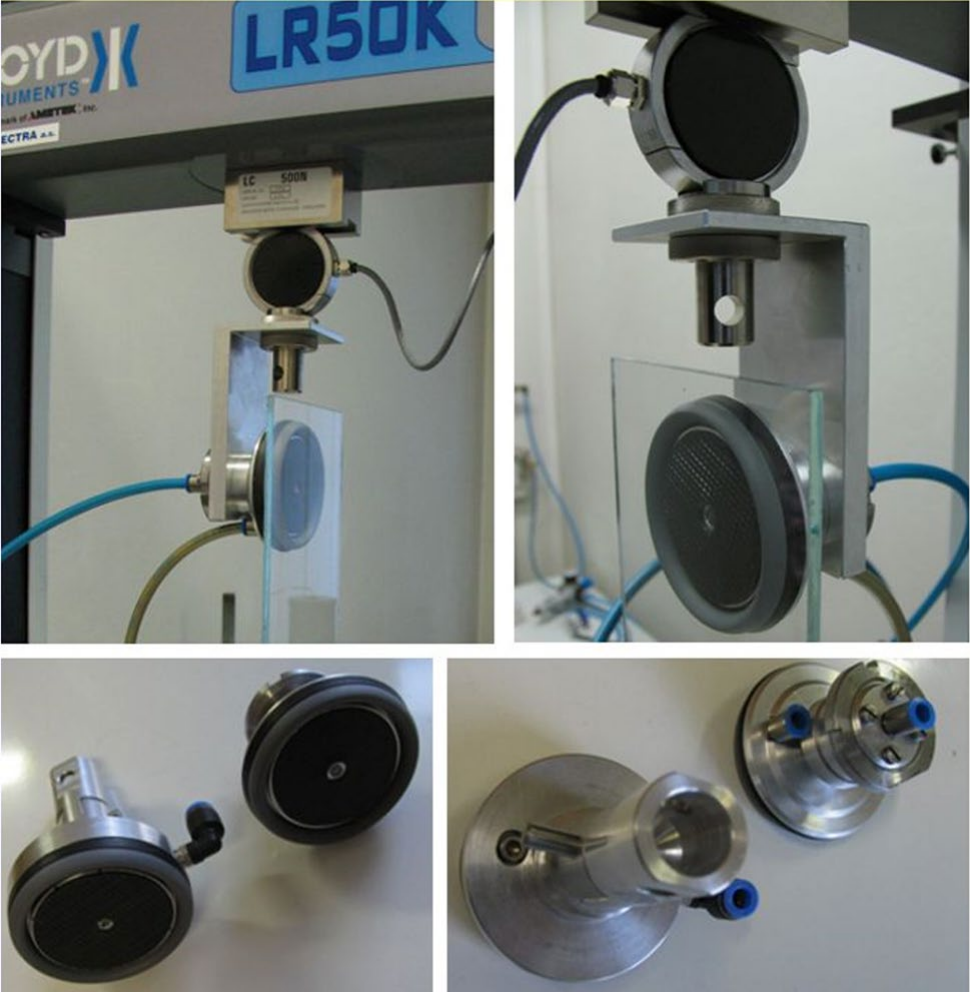
Functional samples of designed gripping elements

Designed gripping elements (Fig. 31) were prepared as functional samples and their function was tested both under laboratory and subsequently under real operational conditions in flat glass production. Realized tests validated presumptive characteristics and confirmed their declared advantages compared to standardly available solutions of gripping elements. It is possible to state, that in comparison with biologically inspired GSA adhesive materials these proposed gripping elements have higher lifespan, mechanical resistance and better availability.

## Conclusion

To increase the gripping safety and loading capacity of commonly used vacuum gripping elements (suction cups), an analysis of the application of new materials with a high degree of surface adhesion in the design of the gripping heads was performed. For this, a theoretical description of the contact including adhesion and cohesion zones was prepared. The performed experiments made it possible to determine the critical cohesive energy for defined samples of a PU-based adhesive material (gel), which was expressed in a unique way for the needs of computer modeling—non-linear time dependence. This made it possible to representatively model the relatively complicated behavior of the adhesive contact in the design of combined gripping elements.

The combined elements represent an interesting alternative to the standard vacuum suction cups, and significantly increase the loading capacity (gripping force), while maintaining a similar geometry. In the framework of the presented research, a unique principle was designed, and a combined element was subsequently designed, which works in both an active and completely passive mode without the need for an energy source, which is beneficial for e.g., manipulation in a vacuum.

The designed element has an adhesive layer with controlled pressure, which has a fundamental influence on the formation, geometry, and qualitative character of the cohesion zones. The solution includes an active system for disturbing the gripping force and a specifically designed hydraulic position compensation module, which allows parallel installation of elements and their mutual position correction depending on the profile and geometry of the contact plane of the manipulated object.

The main benefits of the presented article may be summarized as follows:

o The introductory part presents the current trends in research, development, and design of new types of grippers and gripping elements applying new types of materials.
o Adhesive materials on the principle of the gecko effect were characterized and experimental studies of industrial use of PU adhesive gels in the design of gripping elements were performed.
o Based on the performed research, the mathematical aspects of the adhesive contact were clearly formulated, and the dependence of cohesive energy on time expressed by a nonlinear regression curve in an exponential form was determined for the tested material in accordance with the performed experiments.
o A computer model of the behavior of the adhesive layer depending on time, load, and the initial geometric distribution of the contact zone was designed and verified.
o Several application examples of both combined and purely passive gripping elements using PU materials (gels) with a high degree of surface adhesion were conceived and pilot tested.
o For gripping heads with parallel installed adhesive elements, a completely new position compensation module was designed and presented, ensuring the even loading of the individual elements.

## Data Availability

Not applicable.
